# Supplementary material for: Timing Matters: A Systematic Review of Early Versus Delayed Palliative Care in Advanced Cancer
Source: Cancers (Basel). 2025 Aug 7;17(15):2598. doi: 10.3390/cancers17152598 (PMC12346342; doi:10.3390/cancers17152598)
Supplement: Supplementary file 1 [file cancers-17-02598-s001.zip › cancers-3780866-supplementary.pdf]

*Systematic Review*

# Timing Matters: A Systematic Review of Early Versus Delayed Palliative Care in Advanced Cancer-SUPLEMENTARY MATERIAL

**Ioana Creangă-Murariu [1,2,3], Eliza-Maria Froicu [4*], Dragos V. Scripcariu [5], Gema Bacoanu [4,6], Mihaela Poroch [7], Mihaela Moscalu [7], Claudia Cristina Tarniceriu [8], Teodora Alexa-Stratulat [1,2], Vladimir Poroch [4,6]**

[1] Advanced Research and Development Center for Experimental Medicine (CEMEX), Iasi, Romania;

[2] Medical Oncology-Radiotherapy Department, "Grigore T. Popa" University of Medicine and Pharmacy, Iasi, Romania;

[3] Centre for Translational Medicine, Semmelweis University, Budapest, Hungary;

[4] 2nd Internal Medicine Department, Faculty of Medicine, "Grigore T. Popa" University of Medicine and Pharmacy, Iasi, Romania;

[5] Department of Surgery, "Grigore T. Popa" University of Medicine and Pharmacy, Iasi, Romania

[6] Department of Palliative Care, Regional Institute of Oncology, Iasi, Romania;

[7] Department of Preventive Medicine and Interdisciplinarity, "Grigore T. Popa" University of Medicine and Pharmacy, Iasi, Romania.

[8] Department of Morpho-Functional Sciences I, Discipline of Anatomy, "Grigore T. Popa" University of Medicine and Pharmacy, Iasi, Romania.

* Correspondence: eliza-maria.froicu@umfiasi.ro

**Citation:** To be added by editorial staff during production.

Academic Editor: Firstname Lastname

Received: date
Revised: date
Accepted: date
Published: date

**Copyright:** © 2025 by the authors. Submitted for possible open access publication under the terms and conditions of the Creative Commons Attribution (CC BY) license (https://creativecommons.org/licenses/by/4.0/).

| Section and Topic | Item # | Checklist item | Location where item is reported |
|---|---|---|---|
| **TITLE** | | | |
| Title | 1 | Identify the report as a systematic review. | 1 |
| **ABSTRACT** | | | |
| Abstract | 2 | See the PRISMA 2020 for Abstracts checklist. | 1 |
| **INTRODUCTION** | | | |
| Rationale | 3 | Describe the rationale for the review in the context of existing knowledge. | 2 |
| Objectives | 4 | Provide an explicit statement of the objective(s) or question(s) the review addresses. | 2 |
| **METHODS** | | | |
| Eligibility criteria | 5 | Specify the inclusion and exclusion criteria for the review and how studies were grouped for the syntheses. | 3 |
| Information sources | 6 | Specify all databases, registers, websites, organisations, reference lists and other sources searched or consulted to identify studies. Specify the date when each source was last searched or consulted. | 3 |
| Search strategy | 7 | Present the full search strategies for all databases, registers and websites, including any filters and limits used. | 3 |
| Selection process | 8 | Specify the methods used to decide whether a study met the inclusion criteria of the review, including how many reviewers screened each record and each report retrieved, whether they worked independently, and if applicable, details of automation tools used in the process. | 3 |
| Data collection process | 9 | Specify the methods used to collect data from reports, including how many reviewers collected data from each report, whether they worked independently, any processes for obtaining or confirming data from study investigators, and if applicable, details of automation tools used in the process. | 4 |
| Data items | 10a | List and define all outcomes for which data were sought. Specify whether all results that were compatible with each outcome domain in each study were sought (e.g. for all measures, time points, analyses), and if not, the methods used to decide which results to collect. | 4 |
| | 10b | List and define all other variables for which data were sought (e.g. participant and intervention characteristics, funding sources). Describe any assumptions made about any missing or unclear information. | 4 |
| Study risk of bias assessment | 11 | Specify the methods used to assess risk of bias in the included studies, including details of the tool(s) used, how many reviewers assessed each study and whether they worked independently, and if applicable, details of automation tools used in the process. | 5 |
| Effect measures | 12 | Specify for each outcome the effect measure(s) (e.g. risk ratio, mean difference) used in the synthesis or presentation of results. | NA |
| Synthesis methods | 13a | Describe the processes used to decide which studies were eligible for each synthesis (e.g. tabulating the study intervention characteristics and comparing against the planned groups for each synthesis (item #5)). | 4 |
| | 13b | Describe any methods required to prepare the data for presentation or synthesis, such as handling of missing summary statistics, or data conversions. | NA |
| | 13c | Describe any methods used to tabulate or visually display results of individual studies and syntheses. | 4 |
| | 13d | Describe any methods used to synthesize results and provide a rationale for the choice(s). If meta-analysis was performed, describe the model(s), method(s) to identify the presence and extent of statistical heterogeneity, and software package(s) used. | NA |
| | 13e | Describe any methods used to explore possible causes of heterogeneity among study results (e.g. subgroup analysis, meta-regression). | NA |
| | 13f | Describe any sensitivity analyses conducted to assess robustness of the synthesized results. | NA |
| Reporting bias assessment | 14 | Describe any methods used to assess risk of bias due to missing results in a synthesis (arising from reporting biases). | NA |

| Section and Topic | Item # | Checklist item | Location where item is reported |
|---|---|---|---|
| Certainty assessment | 15 | Describe any methods used to assess certainty (or confidence) in the body of evidence for an outcome. | NA |
| **RESULTS** | | | |
| Study selection | 16a | Describe the results of the search and selection process, from the number of records identified in the search to the number of studies included in the review, ideally using a flow diagram. | 5 |
| | 16b | Cite studies that might appear to meet the inclusion criteria, but which were excluded, and explain why they were excluded. | 5 |
| Study characteristics | 17 | Cite each included study and present its characteristics. | 5-6 |
| Risk of bias in studies | 18 | Present assessments of risk of bias for each included study. | 7-9 |
| Results of individual studies | 19 | For all outcomes, present, for each study: (a) summary statistics for each group (where appropriate) and (b) an effect estimate and its precision (e.g. confidence/credible interval), ideally using structured tables or plots. | NA |
| Results of syntheses | 20a | For each synthesis, briefly summarise the characteristics and risk of bias among contributing studies. | 7-9 |
| | 20b | Present results of all statistical syntheses conducted. If meta-analysis was done, present for each the summary estimate and its precision (e.g. confidence/credible interval) and measures of statistical heterogeneity. If comparing groups, describe the direction of the effect. | 7-13 |
| | 20c | Present results of all investigations of possible causes of heterogeneity among study results. | NA |
| | 20d | Present results of all sensitivity analyses conducted to assess the robustness of the synthesized results. | NA |
| Reporting biases | 21 | Present assessments of risk of bias due to missing results (arising from reporting biases) for each synthesis assessed. | 7-9 |
| Certainty of evidence | 22 | Present assessments of certainty (or confidence) in the body of evidence for each outcome assessed. | NA |
| **DISCUSSION** | | | |
| Discussion | 23a | Provide a general interpretation of the results in the context of other evidence. | 14-15 |
| | 23b | Discuss any limitations of the evidence included in the review. | 15 |
| | 23c | Discuss any limitations of the review processes used. | 15 |
| | 23d | Discuss implications of the results for practice, policy, and future research. | 15 |
| **OTHER INFORMATION** | | | |
| Registration and protocol | 24a | Provide registration information for the review, including register name and registration number, or state that the review was not registered. | 3 |
| | 24b | Indicate where the review protocol can be accessed, or state that a protocol was not prepared. | 3 |

| Section and Topic | Item # | Checklist item | Location where item is reported |
|---|---|---|---|
| | 24c | Describe and explain any amendments to information provided at registration or in the protocol. | NA |
| Support | 25 | Describe sources of financial or non-financial support for the review, and the role of the funders or sponsors in the review. | 16 |
| Competing interests | 26 | Declare any competing interests of review authors. | 16 |
| Availability of data, code and other materials | 27 | Report which of the following are publicly available and where they can be found: template data collection forms; data extracted from included studies; data used for all analyses; analytic code; any other materials used in the review. | 16 |

**Supplementary Table S2.** PRISMA Checklist.

| Author, year | Title | Reason for exclusion |
|---|---|---|
| Zimmermann et al 2019 | Phase 2 trial of symptom screening with targeted early palliative care (step) for patients with advanced cancer | Conference abstract |
| Singhai et al, 2019 | Effect of early integration of specialized palliative care into standard oncologic treatment on the quality of life of patients with advanced head and neck cancers | Conference abstract |
| Sinnarajah et al, 2019 | Palliative care early and systematic (paces): Assessing patient and caregiver preferences for early palliative care delivery in rural Alberta | Conference abstract |
| Dhollander et al, 2019 | Feasibility and acceptability of the early palliative home care embedded in cancer treatment (ephect) intervention | Conference abstract |
| Bendiane et al, 2019 | Under prescription/dispensing of step 3 opioids in french painful cancer survivors: advocacy for integration of early palliative care in oncology. | Conference abstract |
| Greer et al, 2024 | Comparative effectiveness trial of early palliative care delivered via telehealth versus in person among patients with advanced lung cancer. | Conference abstract |
| Linklater et al, 2023 | Early Palliative Care among Older People with Non-malignant Chronic Conditions: A Randomized Controlled Trial | Conference abstract |

| | | |
|---|---|---|
| Niemeyer-Guimarães et al, 2023 | Shared Decision-making in Feeding Strategy for Late-stage Dementia Patients Living in Long-term Assisted-living Institutions for the Elderly (LT/AL): Integration of Early Palliative Care (PC) | Conference abstract |
| Verne et al, 2023 | Palliative Care Should Be Offered to More Liver Disease Patients Earlier as Parallel Planning as their Risk of Death in Each Admission Is Very High | Conference abstract |
| Yang et al, 2023 | Effect of Proactive Monitoring Using the Integrated Palliative Care Outcomes Scale (IPOS) on Patient Quality of Life - A Randomized Controlled Trial | Conference abstract |
| Philip et al, 2023 | Care Plus-integrating Early Palliative Care with Multiple Myeloma | Conference abstract |
| Prod'Homme et al, 2023 | Collaboration and Early Palliative Care after Immune Therapy Revolution in Metastatic Melanoma: A Focus Group Study | Conference abstract |
| Ryan et al, 2023 | Introducing Early Palliative Care to an Integrated Specialist Palliative Care Model in Ireland | Conference abstract |
| Scarpi et al, 2018 | Systematic vs. on-demand early palliative care in gastric cancer patients: a randomized clinical trial assessing patient and healthcare service outcomes | Duplicate |
| Slama et al, 2020 | Effects of Early and Systematic Integration of Specialist Palliative Care in Patients with Advanced Cancer: Randomized Controlled Trial PALINT | Duplicate |
| Bayly et al, 2023 | Integrated Short-term Palliative Rehabilitation to improve quality of life and equitable care access in incurable cancer (INSPIRE): a multinational European research project | Study protocol |
| Bagaragaza et al, 2023 | Assessing the implementation and effectiveness of early integrated palliative care in long-term care facilities in France: an interventional mixed-methods study protocol | Study protocol |
| Webb et al, 2019 | Integration of Palliative Care into Acute Myeloid Leukemia Care | Review article |

**Table S3.** Excluded articles after full-text assessment.
